# Supplementary figures and images for: Evaluating COI and ITS2 dual barcoding for molecular delimitation and taxonomic insights in Arenosetella Wilson, 1932 (Harpacticoida: Ectinosomatidae) along Turkish Coasts
Source: PeerJ. 2025 Aug 21;13:e19870. doi: 10.7717/peerj.19870 (PMC12375299; doi:10.7717/peerj.19870)

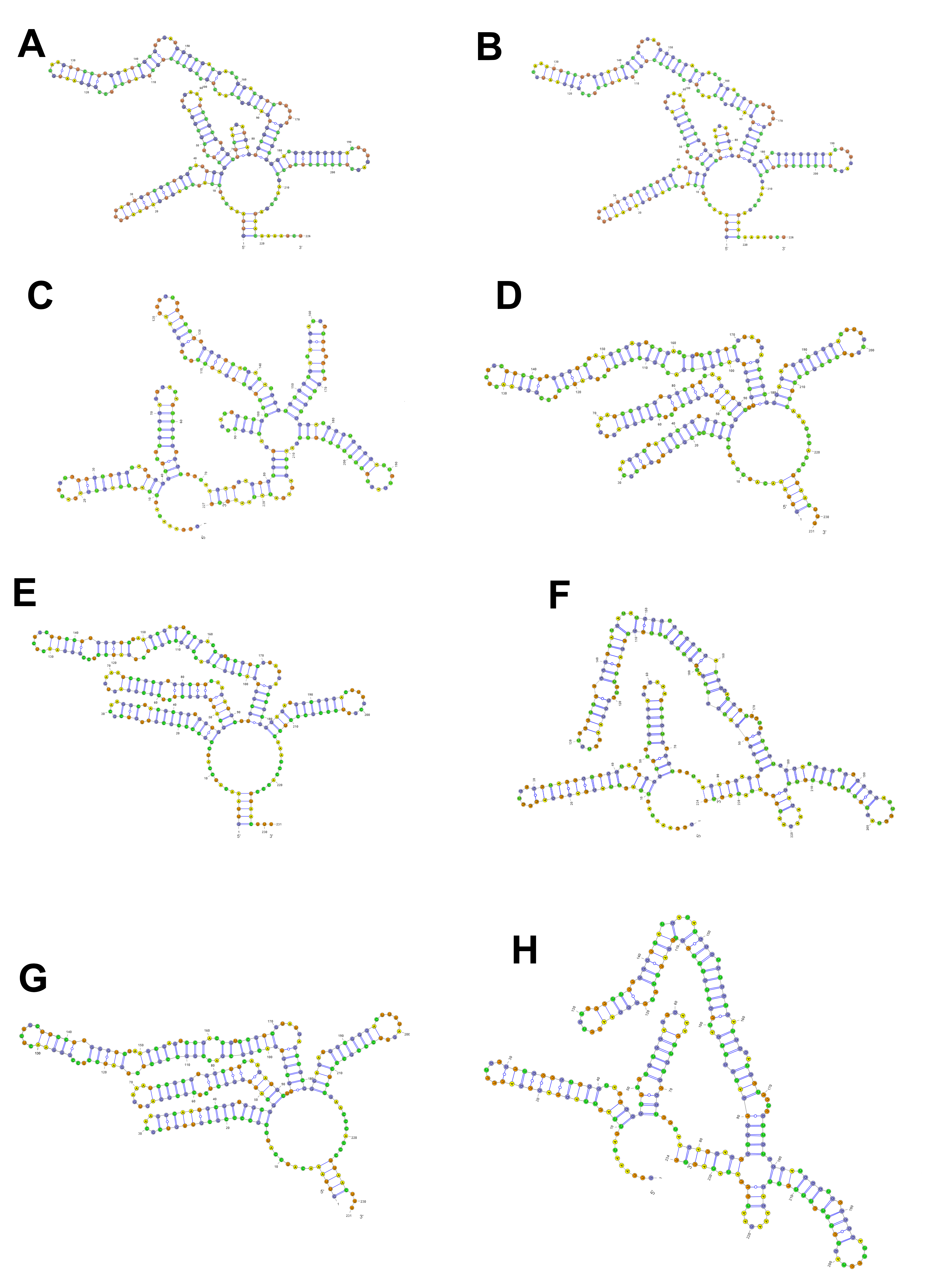

Supplement: Supplemental Information 5 — (A) ITS2 helix structures of Set_1, (B) ITS2 helix structures of Set_2, (C) ITS2 helix structures of Set_4, (D) ITS2 helix structures of Set_5, (E) ITS2 helix structures of Set_6, (F) ITS2 helix structures of Set_7, (G) ITS2 helix structures of Set_8, (H) ITS2 helix structures of Set_9. [file peerj-13-19870-s005.png]
