# Supplementary material for: Evaluating COI and ITS2 dual barcoding for molecular delimitation and taxonomic insights in Arenosetella Wilson, 1932 (Harpacticoida: Ectinosomatidae) along Turkish Coasts
Source: PeerJ. 2025 Aug 21;13:e19870. doi: 10.7717/peerj.19870 (PMC12375299; doi:10.7717/peerj.19870)

```
      (((((.((((...(((((((((((.(((....))).)))))).)))))..((((((.(.(
Set_7 GUUAAACAUCCAUCCAGUAGUGAUUUC-------GUGUCAUU-GCUGGCAUUGGGCUCUC 52
Set_9 GUUAAACAUCCAUCCAGUAGUGAUUUC-------GUGUCGUU-GCUGGCAUUGGGCUCUC 52
Set_4 GUUAAACAUCCAUCCAGCGAUACU-------------GCUUU-GCUGGCAUUGGGCUCUC 46
Set_2 GUUAAACAUCCAUCCAGUGAUAAUUU---------UAUUGUC-GCUGGCAUUGGGCUCUC 50
Set_1 GUUAAACAUCCAUCCAGUGAUAAUUU---------UAUUGUC-GCUGGCAUUGGGCUCUC 50
Set_3 GUUAAACAUCCAUCCGCCCCGCGUUGUCGCAAGGCAAUGCGGUGGCGGCAUUGGGCUCUC 60
Set_8 GUUAAACAUCCAUCCGCCCCGCGUUGUCGCAAGGCAAUGCGGUGGCGGCAUUGGGCUCUC 60
Set_5 GUUAAACAUCCAUCCGCCCCGCGUUGUCGCAAGGCUACGCGGUGGCGGCAUUGGGCUCUC 60
Set_6 GUUAAACAUCCAUCCGCCCCGCGUUGUCGCAAGGCUACGCGGUGGUGGCAUUGGGCUCUC 60
      .........10........20........30........40........50........6

      (((((......)))))).))).))))))))..((((((((((.((...((((((((((..
Set_7 GCGGAGUGAAAUCCGCGUGGCUUCAAGAUGAGUGCGGUGACGGCCGACUGCCGCCCUGCU 112
Set_9 GCGGAGUGAAAUCCGCGUGGCUUCAAGAUGAGUGCGGUGACGGCCGACUGCCGCCCUGCU 112
Set_4 GCGGAGUGACAUCCGCGUGGCUUCAAGAUGAGUGCGGUGACGGCCGACUGCCGCCCUGCU 106
Set_2 GCGGAGUGAAAUCCGCGUGGCUUCAAGAUGAGUGCGGUGACGGCCAACUGCCGCCCUGCU 110
Set_1 GCGGAGUGAAAUCCGCGUGGCUUCAAGAUGAGUGCGGUGACGGCCAACUGCCGCCCUGCU 110
Set_3 GCGGAGUGAAAUCCGCGUGGCUUCAAGAUGCUUGCGGUGACGGCG-ACCGG--CCGCUCU 117
Set_8 GCGGAGUGAAAUCCGCGUGGCUUCAAGAUGCUUGCGGUGACGGCG-ACCGG--CCGCUCU 117
Set_5 GCGGAGUGAAAUCCGCGUGGCUUCAAGAUGCUUGCGGUGACGGCG-ACCGG--CCGCUCU 117
Set_6 GCGGAGUGAAAUCCGCGUGGCUUCAAGAUGCUUGCGGUGACGGCG-ACCGG--CCGCUCU 117
      0........70........80........90........100.......110.......1

      (((((...(((((......))))).)))))......)))).))))))..))..))))...
Set_7 GAUCCUCUGGGAAUUCGCUUUCCUUGGGUCACGACACGGGCGCGGCGACGGGACGUC--U 170
Set_9 GAUCCUCUGGGAAUUCGCUUUCCUUGGGUCACGACACGGGCGCGGCGACGGGACGUC--U 170
Set_4 GAUCCUCUGGGAAUUUGCUUUCCUUGGGUCACGACACGGGCGCGGCGACAGGACGUCUUU 166
Set_2 GAUCCUCCGGGAAUGCAAAUUCCUUGGAUCGCGUUGAUGG-GCGACGACCGGACGUCUUU 169
Set_1 GAUCCUCCGGGAAUGCAAGUUCCUUGGAUCGCGUUGAUGG-GCGACGACCGGACGUCUUU 169
Set_3 GAUCCUCCGGGAACUCUGCUUCCUUGGGUC----UAAGCGUGU-CCGACCGUCCGUCGUA 172
Set_8 GAUCCUCCGGGAACUCUGCUUCCUUGGGUC----UAAGCGAGU-CCGACCGUCCGUCGUA 172
Set_5 GAUCCUCCGGGAACUCUGCUUCCUUGGGUC----UAAGCGAGU-CCGACCGUCCGUCGUA 172
Set_6 GAUCCUCCGGGAACUCUGCUUCCUUGGGUC----UAAGCGAGU-CCGACCGUCCGUCGUA 172
      20.......130.......140.......150.......160.......170.......1

      ...))))))(((.(((...(((((........)))))))))))..........)))))..
Set_7 UCUGCCGCGGGACGGGCGUGGGGACCUUCAGCUCCCUCCCUCCGAGAAAGAACUUUACAC 230
Set_9 UCUGCCGCGGGACGGGCGUGGGGACCUUCAGCUCCCUCCCUCCGAGAAAGAACUUUACAC 230
Set_4 UCUGCCGCGGGACGGGCGGGGA--CCUCGAGCUCCCCCCCUCCGA-ACCGAUAUACACAA 223
Set_2 U-UGCCGCGGGACGGG---GGGGACUUUGAGCUCCCCCUCUCCGA---ACCGAUAACAAA 222
Set_1 U-UGCCGCGGGACGGG---GGGGACUUUGAGCUCCCCCUCUCCGA---ACCGAUAACAAA 222
Set_3 UGAGCCGCGGGA--GAUGGCGGGACGUUGCGUUCCCGCCCUCCGAACCCACCCUUAAC-- 228
Set_8 UGAGCCGCGGGA--GAUGGCGGGACUUUUAGUUCCCGCCCUCCGAACCCACCCUUAAC-- 228
Set_5 UGAGCCGCGGGA--GAUGGCGGGACUUUGCGUUCCCGCCCUCCAAACCCACCCUUAAC-- 228
Set_6 UGAGCCGCGGGA--GAUGGCGGGACUUUGCGUUCCCGCCCUCCAAACCCACCCUUAAC-- 228
      80.......190.......200.......210.......220.......230.......2

      ....
Set_7 AUCU 234
Set_9 AUCU 234
Set_4 AUCU 227
Set_2 AUCU 226
Set_1 AUCU 226
Set_3 -UUU 231
Set_8 -UUU 231
Set_5 -UUU 231
Set_6 -UUU 231
      40..
```

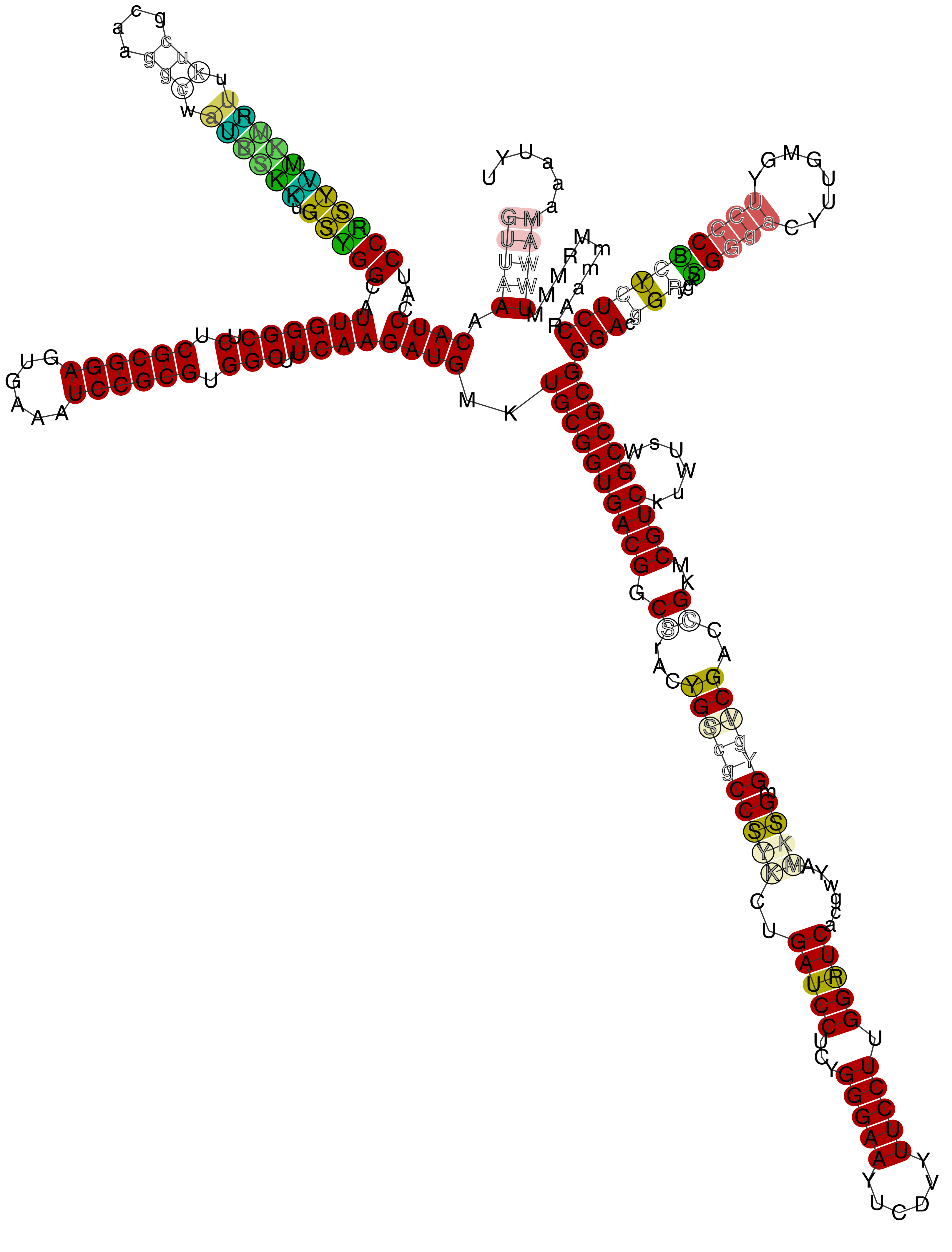

Supplement: Supplemental Information 4 [file peerj-13-19870-s004.pdf]
